# Production of single cell oil from cane molasses by *Rhodotorula kratochvilovae* (syn, *Rhodosporidium kratochvilovae*) SY89 as a biodiesel feedstock

**DOI:** 10.1186/s13065-018-0457-7

**Published:** 2018-08-10

**Authors:** Tamene Milkessa Jiru, Laurinda Steyn, Carolina Pohl, Dawit Abate

**Affiliations:** 1 Department of Biotechnology, University of Gondar, P.O.Box: 196, Gondar, Ethiopia; 2 Department of Microbial, Biochemical and Food Biotechnology, University of the Free State, P.O.Box: 339, Bloemfontein, South Africa; 3 Microbial, Cellular and Molecular Biology Department, College of Natural Sciences, Addis Ababa University, P.O.Box: 1176, Addis Ababa, Ethiopia

**Keywords:** Cane molasses, Biodiesel, Oleaginous yeast, Single cell oil, *Rhodotorula kratochvilovae* (syn, *Rhodosporidium kratochvilovae*)

## Abstract

**Background:**

Single cell oil has long been considered an alternative to conventional oil sources. The oil produced can also be used as a feedstock for biodiesel production. Oleaginous yeasts have relatively high growth and lipid production rates, can utilize a wide variety of cheap agro-industrial wastes such as molasses, and can accumulate lipids above 20% of their biomass when they are grown in a bioreactor under conditions of controlled excess carbon and nitrogen limitation.

**Results:**

In this study, *Rhodotorula kratochvilovae* (syn, *Rhodosporidium kratochvilovae*) SY89 was cultivated in a nitrogen-limited medium containing cane molasses as a carbon source. The study aims to provide not only information on the production of single cell oil using *R. kratochvilovae* SY89 on cane molasses as a biodiesel feedstock, but also to characterize the biodiesel obtained from the resultant lipids. After determination of the sugar content in cane molasses, *R. kratochvilovae* SY89 was grown on the optimized cane molasses for 168 h. Under the optimized conditions, the yeast accumulated lipids up to 38.25 ± 1.10% on a cellular dry biomass basis. This amount corresponds to a lipid yield of 4.82 ± 0.27 g/L. The fatty acid profiles of the extracted yeast lipids were analyzed using gas chromatography, coupled with flame ionization detector. A significant amount of oleic acid (58.51 ± 0.76%), palmitic acid (15.70 ± 1.27%), linoleic acid (13.29 ± 1.18%) and low amount of other fatty acids were detected in the extracted yeast lipids. The lipids were used to prepare biodiesel and the yield was 85.30%. The properties of this biodiesel were determined and found to be comparable to the specifications established by ASTM D6751 and EN14214 related to biodiesel quality.

**Conclusions:**

Based on the results obtained, the biodiesel from *R. kratochvilovae* SY89 oil could be a competitive alternative to conventional diesel fuel.

## Background

Oleaginous microorganisms, including yeasts, which are capable of accumulating lipids, have long been considered an alternative to conventional oil sources. Oleaginous yeasts have high growth and lipid production rates, can utilize a variety of waste carbon sources (including cheap agro-industrial residues such as molasses) and can accumulate lipids from 20 to 70% of their dry cell biomass when grown in a bioreactor under conditions of controlled carbon excess and nitrogen limitation [1, 2].

Biodiesel is a biodegradable, nontoxic, environmentally friendly and cleaner fuel alternative to petroleum-derived diesel fuel [3–6]. It has attracted much attention recently because it is made from renewable resources [7] and may reduce net carbon dioxide emissions by 78% on a life cycle basis [8] and hence contributes to the reduction in emissions to global warming [9].

Biodiesel is currently produced from plant oils and/or animal fats by transesterification with short chain or low molecular weight alcohols such as methanol [6, 10–12]. However, producing biodiesel from vegetable oils or animal fats has many limitations. Firstly, it competes with the food market, since these oils and fats are also used for human consumption. Secondly, using oils, especially vegetable oils, as raw materials have high costs. Thirdly, more time and man power are needed for their production [4, 13]. To compensate this cost, oleaginous microorganisms have to be grown on low cost feedstocks (agro-industrial wastes) and begin to replace the above fats and oil sources. These agro-industrial wastes include molasses, wheat bran, sugar cane bagasse, corn stover, wheat straw, saw mill and paper mill waste [14]. From the many substrates proposed for the economic conversion to lipids, molasses is considered as one of the best feedstocks for the cultivation of lipid producing microorganisms [15]. Molasses is a dark brown viscous liquid obtained as a by-product in the processing of cane or beet sugar. Molasses contains uncrystallized sugar and some sucrose. It is used in the production of bio-polymer [16], bio-surfactant [17], lactic acid [18], bio-ethanol [19–21] and biodiesel [15, 22–24].

Most of the oleaginous yeasts are basidiomycetes. Many basidiomycetous yeasts including *Cryptococcus*, *Trichosporon* and *Rhodosporidium* are now included in other existing or new genera [25]. Accordingly, *Rhodosporidium* has been transferred to *Rhodotorula* and the oleaginous yeast *Rhodosporidium kratochvilovae* is renamed as *Rhodotorula kratochvilovae* [25].

Although other substrates have been investigated as medium for lipid production by this yeast [26], this study aims to provide not only information on the production of single cell oil using the oleaginous yeast, *R. kratochvilovae* SY89 on cane molasses as a biodiesel feedstock, but also to characterize the biodiesel obtained from the resultant lipids.

## Methods

### Yeast strain

In this study, 200 samples were collected from soil, plant surfaces (leaves, flowers and fruits), traditional oil mill wastes, and dairy products (cheese, milk and yoghurt) in Ethiopia. Three hundred and forty yeast colonies were isolated from these samples. It was found that the yeast strain SY89, which was isolated from soil contained oil content of 39.33 ± 0.57% w/w. For identification purposes both conventional (morphological and physiological) and molecular (sequencing both ITS domains and D1/D2 domains of the large subunit) methods were undertaken by Jiru et al. [27]. Identification results led to assign strain SY89 as *R. kratochvilovae*.

### Inoculum preparation

A pre-inoculum was prepared by taking a loopful of yeast cells from growing on slants of Yeast Malt (YM) extract agar (glucose 10 g/L, peptone 5 g/L, yeast extract 3 g/L, malt extract 3 g/L and agar 20 g/L). This was inoculated into a sterilized nitrogen-limited medium containing [glucose 50 g/L, (NH_4_)_2_SO_4_ 0.31 g/L, yeast extract 0.50 g/L, MgSO_4_·7H_2_O 1.5 g/L, CaCl_2_·2H_2_O 0.1 g/L, KH_2_PO_4_ 1.0 g/L, FeSO_4_·7H_2_O 0.035 g/L, ZnSO_4_·7H_2_O 0.011 g/L, MnSO_4_·H_2_O 0.007 g/L, CoCl_2_·6H_2_O 0.002 g/L, Na_2_MoO_4_·2H_2_O 0.0013 g/L and CuSO_4_·5H_2_O 0.001 g/L]. The culture was allowed to grow for 24 h at 30 °C, pH 5.5 at 200 rpm. From this culture, an inoculum of 10% v/v (~ 7.94 × 10^8^ cells/mL) was added to the fermentation medium.

### Bioreactor cultivation using molasses as a substrate

Molasses was used as a carbon source in the cultivation medium for this oleaginous yeast. The molasses was obtained from Wonji Sugar Factory, Wonji, Ethiopia. It was diluted to 50% (v/v). The diluted molasses was then boiled, allowed to cool and sedimentation of insoluble materials occurred. The sediments were removed by decantation. The resulting molasses was centrifuged at 5000×*g* for 10 min for further removal of insoluble materials. The supernatant was separated from the pellet. The pellet was discarded and the supernatant was used for the cultivation purpose. Glucose, fructose and sucrose contents of the molasses were determined by HPLC (Waters Corp., Milford, MA, USA) using an Aminex HPX-87P column (300 × 7.8 mm) at 85 °C with MilliQ water at a flow rate of 0.6 mL/min as eluent. The injection volume was 10 μL. Peak identification of each sugar was based on the retention times (tR) of each sugar [sucrose (tR = 17.45 min), glucose (tR = 21.98 min) and fructose (tR = 25.96 min)]. Before the quantitative determination of sugars in the molasses, standard solutions of sucrose, glucose and fructose were prepared and used to prepare calibration curves for each sugar. The concentrations of the different sugars in the molasses were determined using these curves. The fermentation medium [Molasses 13.10% v/v (~ 50 g/L total sugar), (NH_4_)_2_SO_4_ 0.31 g/L, yeast extract 0.50 g/L, MgSO_4_·7H_2_O 1.5 g/L, CaCl_2_·2H_2_O 0.1 g/L, KH_2_PO_4_ 2.0 g/L, FeSO_4_·7H_2_O 0.035 g/L, ZnSO_4_·7H_2_O 0.011 g/L, MnSO_4_·H_2_O 0.007 g/L, CoCl_2_·6H_2_O 0.002 g/L, Na_2_MoO_4_·2H_2_O 0.0013 g/L, and CuSO_4_·5H_2_O 0.001 g/L] was autoclaved, inoculated with 10% (v/v) of the liquid inoculum and cultivated in a FerMac 320, 0.8 L stirred-tank bioreactor. Fermentations were performed under the following optimized conditions [28]: work volume: 0.6 L, stirring rate: 500 rpm, culture temperature, 30 °C, initial pH, 5.5, aeration rate: 1.5 vvm and culture time, 168 h.

### Cell dry weight determination

Yeast cells were harvested by centrifugation at 5000×*g* for 15 min, washed twice with distilled water, frozen at − 80 °C and freeze dried overnight to constant weight. The dry biomass was determined gravimetrically [6].

### Determination of lipid content

Lipid extraction was done following the protocol described by Folch et al. [29], with some modifications. Freeze dried biomass was ground with a pestle and mortar and 1 g of sample was extracted with 3.75 mL solvent mixture of chloroform and methanol (2:1) overnight. The solvent mixture was filtered (Whatman No 1 filter paper) into a clean separating funnel followed by the addition of 1.25 mL of the solvent mixture. The extract was washed with 0.75 mL of distilled water. The solvent/water mixture was left overnight to separate into two clear phases. The bottom phase was collected and the solvent mixture was evaporated under vacuum. Diethyl ether was used to transfer the extract into pre-weighed glass vials and the solvent evaporated. The dry lipids were weighed and lipid content calculated.

$${\text{Single cell oil content (\% )}} = \frac{\text{Single cell oil weight (g/L)}}{\text{Cell dry weight (g/L)}} \times 100$$

### Analysis of fatty acids profiles using gas chromatography

To determine the fatty acid composition of the lipids, the extracted lipids were dissolved in chloroform, transferred to GC vials and methylated with trimethylsulphonium hydroxide [30]. The vials were then sealed and vortexed for approximately 5 s. Fatty acid methyl esters were subsequently analyzed on a Shimadzu GC-2010 gas chromatograph with a flame ionization detector. An injection volume of 0.5 µL of sample was added into a SGE-BPX-70 column (length of 50 m and inner diameter 0.22 mm). The injection port had a temperature of 250 °C and a split ratio of 1:10. The column temperature was 200 °C. Hydrogen gas was used as a carrier gas at a flow rate of 40 mL/min. The total program time was 4.50 min per sample with a column flow rate of 1.37 mL/min. Peaks were identified by reference to authentic standards.

### Conversion of single cell oil into biodiesel

After extraction of the microbial lipids, sulfuric acid catalyzed transesterification was performed in a 100 mL round bottom flask under the following conditions [31]: reaction time, 7 h; agitation speed, 200 rpm; temperature, 55 °C; oil and methanol molar ratio, 12:1 and catalyst, 0.25 mL of 80% H_2_SO_4_. Petroleum ether was used to separate the biodiesel (upper) layer. The reaction mixture was cooled undisturbed and set aside for phase separation. The final product biodiesel was obtained after evaporating the ether solution. Biodiesel yield (wt%) relative to the weight of the yeast lipid was calculated [31].

$${\text{Biodiesel yield (\% )}} = \frac{\text{Mass of biodiesel}}{\text{Theoretical mass}} \times 100$$

### Characterization of biodiesel properties

The different properties of biodiesel produced from the oil extracted from *R. kratochviolovae* SY89 was calculated directly from the FAME (fatty acid methyl ester) profiles using the online version of Biodiesel Analyzer Software (Biodiesel Analyzer© Version, 2.2.,2016, http://www.brteam.ir/analysis/). The fuel properties of biodiesel analyzed include saponification value (SV), iodine value (IV), cetane number (CN), cloud point (CP), density (ρ), kinematic viscosity (υ), oxidation stability (OS), pour point (PP), cold filter plugging point (CFPP), long chain saturated factor (LCSF), high heating value (HHV), saturated fatty acid (SFA), monounsaturated fatty acid (MUFA), polyunsaturated fatty acid (PUFA), degree of unsaturation (DU), allylic position equivalent (APE) and bis-allylic position equivalent (BAPE).

### Statistical analysis

All experiments were done in triplicate. One way-ANOVA was performed to calculate significant differences in treatment means. SPSS version 20.0 software was used for interpretation of the data. Mean separations were performed by Tukey post hoc tests. A *p* value < 0.05 was considered significant.

## Results and discussion

### Bioreactor cultivation using molasses as a substrate

In this study, single cell oil production from cane molasses by *R. kratochvilovae* SY89 was developed for the first time. Prior to cultivation of *R. kratochvilovae* SY89, the concentrations of the three sugars present in cane molasses were determined using HPLC. The concentration of glucose, fructose and sucrose in molasses is presented in Table 1. The estimated total sugar, calculated as the sum of the three sugars, was 38.28%.

**Table 1:** Composition of sugars in cane molasses

Sugars	Composition in cane molasses (%)
Fructose	21.05
Glucose	15.94
Sucrose	1.29
Total	38.28

**Fig. 1:**
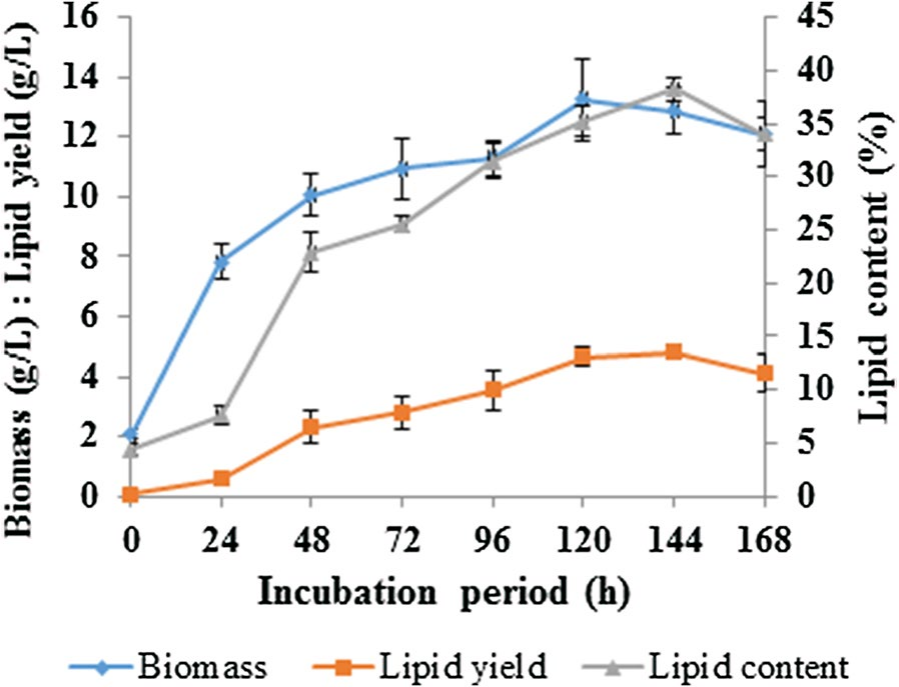
Time course of biomass production, lipid yield and lipid content by *R. kratochvilovae* SY89 using molasses as a substrate in stirred tank bioreactor. Error bars in the figures represent standard deviation

After determination of sugar content in cane molasses, *R. kratochvilovae* SY89 was grown on the optimized cane molasses for 168 h. Under these conditions, this yeast was able to accumulate lipids up to 38.25 ± 1.10% on a cellular dry biomass basis. This result corresponds to a lipid yield of 4.82 ± 0.27 g/L. This maximum value was obtained at 144 h of incubation. On the other hand, maximum biomass of 13.25 ± 1.36 g/L was achieved at 120 h of incubation (Fig. 1).

Previous studies also reported the use of molasses as a substrate for oleaginous yeasts such as *R. glutinis* [22], *Candida lipolytica*, *C. tropicalis* and *Rhodotorula mucilaginosa* [23], *Geotrichum* (syn, *Trichosporon*) *fermentans* [32], *R. glutinis* CCT 2182, *Rhodotorula* (syn, *Rhodosporidium*) *toruloides* CCT 0783, *R. minuta* CCT 1751 and *Lipomyces starkeyi* DSM 70296 [33] for the production of biomass and hence lipid yield.

### Fatty acid composition

The quality of biodiesel depends upon the fatty acid composition of the oil feedstock. The data obtained in this study revealed that when cane molasses was used as a substrate, the yeast appeared to produce oleic acid as the largest lipid component (58.51 ± 0.76%), followed by palmitic acid (15.70 ± 1.27%), linoleic acid (13.29 ± 1.18%), stearic acid (4.38 ± 0.36%), linolenic acid (2.76 ± 0.97%) and palmitoleic acid (0.59 ± 0.17%). Trace amounts (1.70 ± 0.23%) of other fatty acids were also detected. The relative percentage of saturated and monounsaturated fatty acids of *R. kratochvilovae* SY89 adds up 79.18 ± 2.56% which makes the lipids from this strain a suitable oil feedstock for biodiesel production [34]. Highly unsaturated fatty acids are easily oxidized during long term storage and have negative influence to the engine motor and are not recommended for biodiesel production [35].

Similar results on fatty acid profiles of other oleaginous yeasts grown on molasses were reported by other researchers [33, 36]. Other researchers have also reported the fatty acid compositions of oleaginous yeasts that were grown on other wastes such as hydrolysate of cassava starch [37] and crude glycerol [38]. According to their reports, lipids from these yeasts also contained mainly oleic and palmitic and to a lesser extent linoleic and stearic acids. The fatty acid profiles of *R. kratochvilovae* SY89 were not only similar to fatty acid profiles of other oleaginous yeasts but are similar to the fatty acid profiles of different vegetable oils such as rapeseed, soybean, palm, and sunflower [39, 40].

### Production of biodiesel

To produce microbial biodiesel, the extracted oil from *R. kratochvilovae* SY89 was transesterified using methanol and a yield of 85.30% was obtained. Dai et al. [3] also obtained a biodiesel yield of 81.70% from *R. glutinis* by growing the yeast on lignocellulosic wastes. From a previous study, biodiesel yields of 68% and 63% were obtained from heterotrophic growth of *Chlorella protothecoides* at molar ratio levels of 45:1 and 56:1, respectively [31]. From this one can see that the biodiesel yield obtained in this study is better than previous work.

### Characterization of biodiesel properties

To evaluate the potential of biodiesel produced from *R. kratochvilovae* SY89 as a substitute for diesel fuel, the different physico-chemical properties were determined. As shown in Table 2, the results were compared with US biodiesel standard, ASTM D6751 [41] and EU biodiesel standard, EN14214 [42]. Iodine value (IV) for the produced yeast biodiesel, which is a measure of degree of unsaturation of a lipidic material, was 84.83 mg I/100 g oil, which is below the maximum value of 120 mg/100 g oil standard of EN14214. The degree of unsaturation greatly influences fuel oxidation tendency. Cetane number (CN) which is dimensionless descriptor and indicator of the combustion speed of diesel fuel is required for good engine performance [43]. It determines the combustion behavior of the biodiesel, i.e., ignition delay time, which is the time between the injection and ignition [44]. Higher CN helps to ensure good cold start properties and minimize the formation of white smoke. The CN recorded in this study was 55.60. This value is in agreement with the standards for biodiesel, which recommend a minimum CN of 47 (ASTM D6751) or 51 (EU biodiesel standard EN14214) [45]. The oxidation stability (OS) value of FAME for the present study was 9.94, which is an important feature related to the stability and performance of biodiesel. This shows the biodiesel produced from *R. kratochvilovae* SY89 oil is stable. The kinematic viscosity (υ) of the biodiesel produced in this study was 3.66 mm^2^/S and therefore falls in the ranges set by both US biodiesel standard ASTM D6751 (1.6–9.0 mm^2^/S) and EU biodiesel standard EN14214 (3.5–5.0 mm^2^/S). The density (ρ) recorded for this biodiesel was 0.83 g/cm^3^, which is approximated to the biodiesel standard of EN14214 (0.86–0.9 g/cm^3^). Both kinematic viscosity (υ) and density (ρ) influence engine performance, combustion and exhaust emissions. A value of − 3.28 °C for pour point (PP) was obtained in this study. This value also falls in the range set by US biodiesel standard ASTM D6751 (− 15 to 10 °C). Cloud point (CP) of 3.27 °C was obtained in this study. The value 3–15 °C is set by US biodiesel standard ASTM D6751. Saponification values (SV) are used to determine adulteration. A high SV of fats and oils is due to high proportion of shorter carbon chain lengths of the fatty acids and suggests that it has low levels of impurities [46]. A high SV of 192.30 mg KOH/g was recorded for *R. kratochvilovae* SY89 oil. The value recorded for long chain saturated factor is used to calculate the cold filter plugging point (CFPP), which is based on the amount of long chain saturated fatty acids (from C16:0) in the oil was − 4.66 °C. The CFPP value is related to the minimum temperature at which the biodiesel can generate clogging and problems in the motor [47]. The heating value of fatty acid esters increases with molecular chain length (with the number of carbon atoms) and decreases with their degree of unsaturation (the number of double bonds). The heating value for the biodiesel from *R. kratochvilovae* SY89 was 37.63 °C. The biodiesel from this yeast oil could therefore be a competitive alternative to conventional diesel fuel. Other chemical and physical values were analyzed, including SFA (saturated fatty acid), MUFA (monounsaturated fatty acid), degree of unsaturation (DU), long chain saturated factor (LCSF), allylic position equivalent (APE) and bis-allylic position equivalent (BAPE) (summarized in Table 2). These characteristics are also important in determining the quality of a given biodiesel. Most of these properties are in agreement with the specifications established by ASTM D6751 and EN14214 related to biodiesel quality.

**Table 2:** Selected physico-chemical properties of biodiesel produced from *R. kratochvilovae* SY89 grown on molasses compared to standard biodiesel specifications

Biodiesel property	Biodiesel from SY89	US biodiesel standard ASTM D6751	EU biodiesel standard EN14214
SV (mg/g)	192.30		
IV mg I/100 g oil	84.83	NS	< 120
CN	55.60	> 47	> 51
CP (°C)	3.27	3–15	− 4
ρ (g/cm^3^)	0.83	NS	0.86–0.9
υ (mm^2^/S)	3.66	1.6–9.0	3.5–5.0
OS (h)	9.94	3 min	6 min
PP (°C)	− 3.28	− 15 to 10	NS
CFPP (°C)	− 4.66	Summer max 0; winter max < − 15	NS
LCSF	3.76	NS	NS
HHV (°C)	37.63	NS	NS
SFA (%)	20.08	NS	NS
MUFA (%)	59.10	NS	NS
PUFA (%)	16.05	NS	NS
DU	91.20	NS	NS
APE	90.61	NS	NS
BAPE	18.81	NS	NS

*NS* non specified

## Conclusions

There are no reports in the literature concerning cultivation using *R. kratochvilovae* with molasses for the production of microbial oil. This study demonstrated that *R. kratochvilovae* SY89 is able to utilize molasses as a carbon source for the production of biomass and hence lipid yield. As such, this study expands the current knowledge in this regard. After pretreatment of molasses and optimization of its sugar concentration, sufficient dry biomass (13.25 ± 1.36 g/L), lipid yield (4.82 ± 0.27 g/L) and lipid content (38.25 ± 1.10%) were obtained in a bioreactor fermentation. Such single cell oil can be transesterified into biodiesel that conforms to international standards for such fuel. Production of microbial oil using cheap substrates such as molasses may be advantageous for countries like Ethiopia, since the cost of purchasing and transportation of petroleum oil can be reduced at least partially.
